# Green synthesis and characterization of bisphosphonate conjugated gold nanoparticle with *Asparagus racemosus* root extract

**DOI:** 10.6026/97320630018160

**Published:** 2022-03-31

**Authors:** Sruthi Harikrishnan, Navaneethan Ramasamy, Rajeshkumar Shanmugam

**Affiliations:** 1 Department of Orthodontics and Dentofacial Orthopaedics, Saveetha dental college and Hospital, Saveetha Institute of Medical and Technical Sciences (SIMATS), Saveetha University Chennai-77, India; 2 Department of Pharmacology, Saveetha Dental College and Hospital, Saveetha Institute of Medical and Technical Sciences (SIMATS) Saveetha University, Chennai - 77, India

**Keywords:** Bisphosphonate, green synthesis, gold nanoparticles

## Abstract

Bisphosphonates improve orthodontic anchorage. More targeted action of this drug can be achieved through its conjugation with gold nanoparticles. Asparagus racemosus is a green edible medicinal plant used in Ayurvedic preparations to treat aging, vigor, immunity, longevity, and skeletal issues. Therefore, it is of interest to report the green synthesized Bisphosphonate conjugated gold nanoparticles with Asparagus racemosus extract and to characterize them.

## Background:

Nanotechnology aims to design, create and control matter in the dimensional range of 1-100nm [1]. The use of materials in these dimensions provides an opportunity to modify various properties such as solubility, diffusivity, blood circulation half-life, drug release characteristics, and immuno-genicity at the level of atomic or biomolecules [2,3]. In general, synthesis of nanoparticles uses high radiation or concentrated reductants and stabilizing agents that are harmful both to the environment and to human health. Green synthesis of nanoparticles had shown to reduce the toxicity of the nanoparticles [4- 7]. Bisphosphonates are increasingly being used to treat a wide range of skeletal disorders like osteoporosis. Bisphosphonates have been shown to enhance orthodontic anchorage in several studies [8- 11]. Gold nanoparticles have several advantages, including the ability to accelerate osteoblast differentiation, inhibit adipose-derived stem cell differentiation, suppress osteoclast formation, and promote bone formation in bone tissue regeneration. When injected into the body, however, GNPs can cause toxicity. As a result, the surface of these particles must be modified to specifically target bone tissue [12-18]. Asparagus racemosus (A. racemosus, Shatavari) is a green edible medical plant used in Ayurvedic preparations to treat aging, vigor, immunity, longevity, and skeletal issues [19-21]. Bisphosphonate conjugated with gold nanoparticles has been shown to provide a more targeted action. Therefore, it is of interest to report the green synthesis and characterization of bisphosphonate conjugated gold nanoparticles with Asparagus racemosus root extract.

## Materials and Methods:

## Green synthesis gold nanoparticles and conjugation of bisphosphonate:

Roots of Asparagus racemosus were dried in an oven at 30°C and ground to a coarse powder. 1gm of available Asparagus racemosus stem powder was boiled at 100 degrees c with 100 ml of distilled water in a beaker. The extract was then filtered using a filter paper to obtain 75ml. To reduce the Au2+ ions, 30 mL of the extract was added to a reaction vessel containing 70 mL of chloroauric acid solution. The nanoparticles were then centrifuged at 1500 rpm for 10 minutes before being redispersed in 20 mL of distilled water. 2mg/ml of Zoledronic acid was added to one part of the sample gold nanoparticle extract and left to stir in a magnetic stirrer overnight.

## Antimicrobial activity:

Biosynthesized nanoparticles are used in a wide range of biomedical applications. Membrane damage is one of the most common causes of nanoparticle antibacterial properties. Using the agar well diffusion method, the green synthesized bisphosphonate conjugated gold nanoparticles were tested against common oral pathogens such as Candida albicans, Enterococcus fecalis, Staphylococcus aureus, and Lacto bacillus. The test organisms (S. mutans and Lactobacillus) were grown in nutrient broth and kept on agar slants for the study. Candida albicans were grown on Rose Bengal agar, which is a yeast-specific medium. Using a sterile cotton swab, the freshly cultured strains were grown and uniformly spread over petri dishes containing MHS agar (Mueller Hinton 2 agar + 5% sheep blood). With the help of a steel borer, agar wells measuring 6.0 mm in diameter were punched into the culture plate containing the test microorganisms 35.A micropipette was used to fill the agar wells with 20μL of different concentrations of nanoparticles (50,100,150g/ml). As a positive control, 20µL of standard antibiotics (Ampicillin) were used. The diameter of the inhibition zone was measured in millimeters after a 24-hour incubation period at 37°C (mm). All of the tests were performed three times.

## Cytotoxicity:

The eggs of brine shrimp are purchased to perform the cytotoxic assay on brine shrimp. The eggs are then kept at a temperature of 28°C. Artificial seawater and a 37°C light source are used to hatch eggs. This method was tested in 15 well plates (Figure 1 - see PDF)the newly hatched Nauplii are selected and transferred to each well using a Pasteur pipette. The Gold nanoparticles with and without Bisphosphonate conjugation were introduced into each of the wells of varying concentrations of 5,10,15,25 µL is added to each well, and the volume is adjusted. After 24 hours, the brine shrimp are removed from the 15 well plates and counted with a magnifying glass. After a 24-hour incubation period, the percentage of dead shrimp in each well is calculated.

The number of motile nauplii was calculated to assess the cytotoxicity of the nanoparticles

Viability was calculated per well using the formula below

Viability (in %) = live brine shrimp after exposure/live brine shrimp before exposure *100% 

## Result and Discussion:

The color change of the reaction mixtures from light yellow to yellow, dark-purple, and dark brown, respectively, could indicate the biosynthesis of Au nanoparticles in the current experiment. In the current experiment, the visual color change from yellow to dark purple was formed in 6 hours. The reduction confirmation of Au++ to Au0 is shown by the solution's color changing from light brown to dark brown. The brown color variation indicates an incomplete reduction of less concentration in the plant extract solution, whereas the formation of dark brown color at high plant extract concentrations revealed a complete reduction reaction. In the presence of incident photons, Au nanoparticles displayed the surface plasmon resonance (SPR) band as a result of the metal's conduction and free band electrons collectively oscillating. The intensity of the SPR band is primarily determined by the nature of the nanoparticles used in the synthesis, as well as their composition Furthermore, UV-vis spectroscopy is a key tool for determining the nature of synthesized Au. The analysis was carried out every one hour to determine the changes. The analysis showed a consistent peak after 1 hr of preparation at 540 was constantly observed after 2 hours of the preparation of the sample (Figure 1a and 1b - see PDF).

The TEM images and EDX spectra of biosynthesized Au nanoparticles showed that the particles are narrow in size and spherical in shape with a diameter in the range of 10-50 nm (Figure 2 -0 see PDF). However, some froth was noticed on the surface of these obtained nanoparticles, which could be attributed to the different types of phytochemicals present in the plant extract. FTIR analysis confirmed the presence of a huge amount of phytochemicals in the plant extract which can prevent the nanoparticles from agglomeration and helps in the production of stable nanoparticles. There was no other defined morphological difference observed in the preparation of Au nanoparticles. Antibacterial activity of biosynthesized bisphosphonate conjugated gold nanoparticle using Asparagus racemosus extract on Staphylococcus aureus, Lactobacillus, and Candida albicans using agar well diffusion method was performed. This method is widely used to evaluate the antimicrobial activity of plants or microbial extracts. It is qualitative, easy to perform, and simple. The agar plate surface was inoculated by spreading a volume of the microbial inoculums over the entire agar surface. Then, a hole with a diameter of 6 mm was punched aseptically with a sterile cork borer or a tip, and a volume of 20 µL of the nanoparticle sample at desired concentration was introduced into the well. Then, agar plates were incubated under suitable conditions depending upon the test micro-organisms. The nanoparticle sample diffuses in the agar medium and inhibits the growth of the microbial strain tested. Four different concentrations of the nanoparticles were studied (25, 50,100,150µg/ml). The diameter of the zone of inhibition increased with an increase in the concentration of the nanoparticles, against bothS. aureus, Lacto bacillus, and Candida albicans. The diameter of the zone of inhibition against Enterococcus fecalis showed no change with the concentration of the nanoparticles. The zones of inhibition (in milli meters) of gold nanoparticles of varying concentrations, against S. aureus, Lactobacillus, and Candida albicans are represented in Table 1(see PDF). Au nanoparticles have good antibacterial activity against Streptococcus mutans (150ug/ml - 26 mm zone of Inhibition), Lactobacillus (150ug/ml - 26 mm zone of Inhibition), and Candida albicans (150ug/ml - 10 mm zone of Inhibition). Cytotoxicity of the prepared nanoparticles was assessed using Brine Shrimp (Artemia salina) Lethality Assay. It has been demonstrated that the early developmental stages of Artemia salina are highly vulnerable to toxins. The lethality was found to be directly proportional to the concentration of the nanoparticles. Gold nanoparticles with and without bisphosphonate conjugation showed a mortality of 10% at 20 and 25 ug/ml (Table 2 - see PDF).

## Conclusion:

We report the green synthesis and characterization of bisphosphonate conjugated gold nanoparticles with asparagus racemosus root extract for potential application in Dental Biology.
